# Outcomes of hip fracture in centenarians: a systematic review and meta-analysis

**DOI:** 10.1007/s41999-023-00866-y

**Published:** 2023-10-04

**Authors:** Diego Agustín Abelleyra Lastoria, Clerin Kulangara Benny, Toby Smith, Caroline Blanca Hing

**Affiliations:** 1 St George’s University London, London, SW17 0RE UK; 2 Medical University of Sofia, Sofia, 1431 Bulgaria; 3 University of Warwick, Coventry, CV4 7HL UK; 4 St George’s University Hospitals NHS Foundation Trust, London, SW17 0RE UK

**Keywords:** Centenarians, Hip fractures, Meta-analysis, Systematic review, Trauma

## Abstract

**Purpose:**

Outcomes of hip fractures in centenarians remain underreported owing to the small number of patients reaching 100 years of age. This review aimed to determine outcomes of hip fracture in centenarians and to identify the most common comorbidities among centenarians with hip fracture to better characterise this population.

**Methods:**

Published and unpublished literature databases, conference proceedings and the reference lists of included studies were searched to the 25th of January 2023. A random-effects meta-analysis was performed. Included studies were appraised using tools respective of study design.

**Results:**

Twenty-three studies (6970 centenarians) were included (retrospective period: 1990–2020). The evidence was largely moderate to low in quality. One-year mortality following a hip fracture was 53.8% (95% CI 47.2 to 60.3%). Pooled complication rate following a hip fracture in centenarians was 50.5% (95% CI 25.3 to 75.6%). Dementia (26.2%, 95% CI 15.7 to 38.2%), hypertension (15.6%, 95% CI 3.4 to 33.1%), and diabetes (5.5%, 95% CI 1.9 to 10.7%) were the most common comorbidities among centenarians with hip fracture.

**Conclusion:**

Hip fractures in centenarians typically involve complex patient presentations with diverse comorbidities. However, the current evidence-base is moderate to low in quality. Effective cross-discipline communication and intervention is suggested to promote treatment outcomes.

## Key summary points

**Aim:**

We aimed to determine outcomes of hip fracture in centenarians.

**Finding:**

One-year mortality following a hip fracture was 53.8% (95% CI 47.2 to 60.3%). Dementia (26.2%, 95% CI 15.7 to 38.2%) and hypertension (15.6%, 95% CI 3.4 to 33.1%) were the most common comorbidities.

**Message:**

Effective cross-discipline communication and intervention is suggested to promote treatment outcomes.

## Introduction

The number of oldest-old is increasing in high income countries [1], with declining mortality in the 80 years and older age group and improved treatments contributing to an increasing number of centenarians in the last decades. The number of centenarians is projected to increase to between 13 and 50 million individuals worldwide during the twenty-first century [2]. Centenarians experience a higher mortality than other older age groups, such as those aged 80–99 years [1].

Older people are susceptible to trauma owing to an increased risk of delirium and dementia [3], incontinence, frailty, impaired vision and drug interactions [4]. In addition, side effects of commonly used drugs with central nervous system effects (such as benzodiazepines and anti-psychotics) can increase risk of falls, as well as orthostatic hypotension. Resulting fractures are the third most common cause of hospitalisation for this population [5]. Fractures in older people can lead to significant disruptions in daily activities and loss of independence. A review of outcomes following hip fracture estimated between 40 and 60% of patients do not recover their pre-fracture level of mobility, while 30 to 60% do not regain their level of independence for basic activities of daily living [6].

Outcomes of hip fracture in centenarians remain comparatively under-explored owing to the small number of patients reaching 100 years of age [7]. This group of patients are at particular risk of poor outcomes following hip fracture. Centenarians have a high comorbidity burden and polypharmacy, resulting in increased surgical risk [8]. Progressively poorer bone quality than younger individuals predisposes the oldest of people to more complex fractures [9].

To the authors’ knowledge, no systematic reviews have been undertaken assessing the outcomes or characterisation of centenarians who have sustained a hip fracture. Understanding hip fracture outcomes of people 100 years and older is required considering the number of centenarians is projected to increase [2]. Though negative outcomes may be expected due to a high comorbidity burden, these are yet to be formally synthesised. Accordingly, this review aimed to determine outcomes of hip fracture in centenarians and to identify the most common comorbidities among centenarians with hip fractures to better characterise this population.

## Methods

The PRISMA 2020 checklist was satisfied in the reporting of this systematic review [10]. The protocol for this systematic review was prospectively registered (PROSPERO Registration: CRD42022377719).

### Study eligibility

Studies were eligible if they reported outcomes of treatment for hip fractures in centenarians (both conservative and surgical), and/or comorbidities in centenarians who sustain a hip fracture. Both full-texts and abstracts were included. Eligible study designs were case series, case–control, cross-sectional and cohort studies, as well as randomised controlled trials. Both retrospective and prospective studies were eligible. Papers not reporting original data such as literature or systematic reviews were excluded, along with case reports and letters to the editor. There was no constraint based on language, publication status or patient demographics. Eligibility assessment was performed independently by two reviewers (DAAL, CKB). Disagreements regarding study eligibility were solved through discussion.

### Search strategy and data extraction

We searched the following electronic databases via OVID: MEDLINE, Global Health, and Embase. Currently registered studies were reviewed using the databases: ISRCTN registry, the National Institute for Health Research Portfolio, the UK National Research Register Archive, the WHO International Clinical Trials Registry Platform, and OpenSIGLE (system for information on grey literature in Europe). Conference proceedings from the European Federation of National Associations of Orthopaedics and Traumatology (EFORT), British Orthopaedic Association (BOA) and British Trauma Society (BTS) were searched. The reference lists of included studies were also searched (backwards-searching). Finally, papers citing the studies included were also reviewed for eligibility (forward-searching).

Database search and data extraction were conducted independently by two reviewers (DAAL, CKB). Searches were conducted twice for quality assurance. The final search was completed on the 25^th^ of January 2023. The search strategy is presented in Appendix 1 and modified for each respective database.

Data were extracted onto a data extraction template. Data extracted included: baseline characteristics including number of patients, treatment received, patient sex, age, study location, fracture type, fracture management, comorbid diseases, mortality and complications.

### Outcomes

All-cause mortality was calculated, including in-hospital mortality, mortality at 1, 3, 6 months, and 1-year post-fracture. At this extreme of age, mortality beyond 1 year was excluded, as it may be unrelated to the hip fracture. Other outcomes included prevalence of comorbidities in centenarians and non-centenarians, and complications (occurrence and rate) over follow-up periods. The latter were not restricted to a specific time after fracture, since no study defined the duration of the observation period for which complications were monitored. Therefore, we assumed these took place before the final follow-up period post-operatively reported in the studies.

### Methodological appraisal

Level of evidence and risk of bias of each included study were evaluated independently by two reviewers (DAAL, CKB). The level of evidence of the studies presented was determined with the March 2009 Oxford Centre for Evidence-Based Medicine: Levels of Evidence (5 = lowest level of evidence, corresponding to case reports; 1a = highest level of evidence, corresponding systematic reviews of randomised controlled trials) [11]. Each study was appraised with an appraisal tool reflecting the study design. Accordingly, tools used included the Institute of Health Economics case series studies quality appraisal checklist [12] and the Downes and Black Tool for cross-sectional studies [13].

### Data analysis

Where sufficient (at least two) and homogeneous studies reported on the same outcome, a random-effects meta-analysis was performed using MetaXL version 5.3 software (EpiGear International Pty Ltd, Wilston, Queensland, Australia). Data on prevalence was presented as weighted prevalence percentage and 95% confidence intervals (CI). Data on continuous outcomes, i.e. Charlson Comorbidities Index (CCI) was presented as weighed mean difference and 95% CI. Statistical heterogeneity was assessed using Cochran’s Q value and Higgins *I*^2^ statistic for each pooled analysis. This was interpreted in accordance with Higgins and Green [14]. Subgroup analysis of surgical management of hip fractures was undertaken, comparing mortality following surgical intervention with the overall cohort mortality (irrespective of treatment received).

## Results

### Search results

**Fig. 1:**
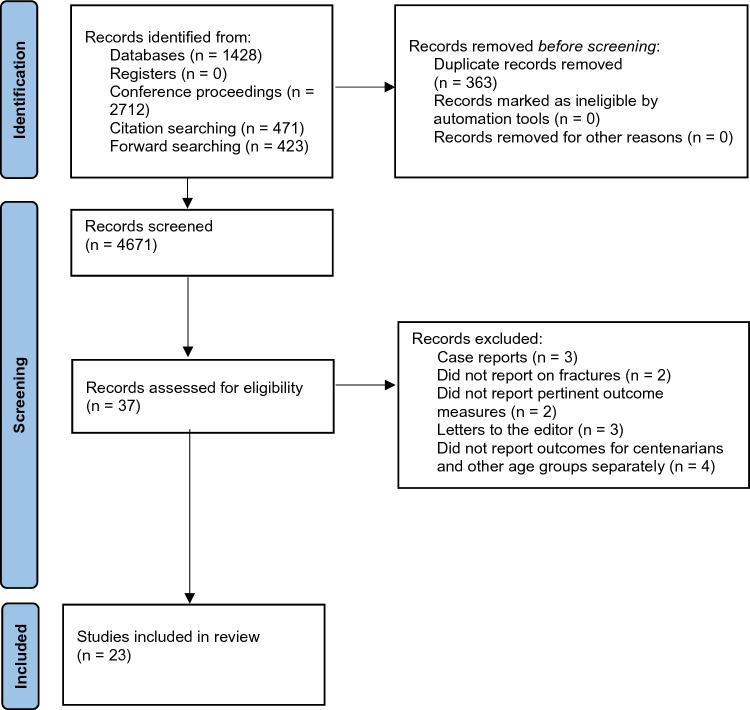
PRISMA diagram depicting the study collection process

In total, 4671 records were screened, of which 23 studies were eligible and evaluated 6970 centenarians (Fig. 1, Table 1). Mean patient age was 101.5 (range 100–111) years. Twenty-two studies reported patient sex (816 males, 11.7% and 6133 females, 88.3%). No study reported whether their patients had concomitant injuries, patient osteoporotic status, nor commented on the severity of hip fracture.

**Table 1:** Baseline characteristics of studies included in meta-analysis

Study	Retrospective period	Number of patients	Patient age (range)/standard deviation	Male/female	Fracture type (number)	Time to surgery (days) (range)/standard deviation	Treatment	Study location	Follow-up duration
Oliver and Burke, 2003 [16]	1998–2002	18	101.8 (100–106)	1/17	NR	NR	Surgical repair	Scotland	4 months
López-Torres et al., 2020 [4]	1999–2018	69	101.3 (100–108)	11/58	Pertrochanteric (43), subcapital (19), basicervical (7)	2	Surgical repair	Spain	1 year
Morice et al., 2017 [25]	2004–2015	39	101.3 (100–108)	6/33	26 extracapsular fractures (9 were A1, 13 were A2, and 4 were A3 in the AO classification). 13 intracapsular (5 were Garden I/II, 8 were Garden III/IV)	1.7 (0–12)	Surgical repair	NR	1 year
Sarasa-Roca et al., 2022 [28]	2009–2019	24	101.1 (100–104)	3/21	Intracapsular (6, 25%), extracapsular (18, 75%)	4.0 ± 2.4	Surgical repair	Spain	1 year
Dick et al., 2017 [32]	2008–2015	23	101.4 (100.2–103.4)	3/19	Intracapsular (13), extracapsular (9), subtrochanteric (1)	1.6 (0.7–6.3)	21 surgical repair, 2 non-operatively	England	5 years
Mazzola et al., 2016 [22]	2004–2011	259	101.5 ± 1.3	25/234	NR	NR	Surgical repair	Italy	2 years
Mosfeldt et al., 2019 [31]	1996–2012	507	101 (100–111)	76/431	Femoral neck fracture (273), pertrochanteric (203), subtrochanteric (31)	NR	NR	Denmark	5 years
Shabat et al., 2004 [17]	1990–2001	23	101.8 (100–107)	6/17	Pertrochanteric (19), subcapital (4)	2.2 ± 0.8	19 Surgical repair, 4 non-operatively	Israel	45 months
Barrett-Lee et al., 2021 [33]	2014–2019	60	101 (100–108)	9/51	NR	NR	56 surgical repair, 4 non-operatively	England	1 year
Moore et al., 2017 [24]	2010–2016	9	101.6 (100–103)	1/8	Neck of femur (3), intertrochanteric (4), subtrochanteric (2)	1.5	Surgical repair	Ireland	28 months
Bermejo Boixareu et al., 2020 [34]	2017–2019	253	NR	42/211	Intracapsular (80), extracapsular (173)	NR	NR	Spain	1 month
Hogan et al., 2019 [45]	2013–2017	57	101 (100–105)	5/52	Intertrochanteric (26), displaced intracapsular (17), other (14)	NR	55 surgical repair, 2 non-operatively	Ireland	NR
Ng and Kwek, 2015 [20]	NR	13	102.3 (100–109)	3/10	Femoral neck, Intertrochanteric and subtrochanteric fractures	NR	6 Surgical repair, 7 non-operatively	NR	1 year
Tarity et al., 2013 [19]	2003–2010	23	102	1/22	NR	NR	21 surgical repair, 2 non-operatively	NR	6 years
Barceló et al., 2018 [30]	NR	33	NR	NR	NR	NR	NR	Spain	1 year
Cheung et al., 2017 [23]	2010–2013	114	(100–109)	18/96	NR	NR	Surgical repair	Hong Kong	1 year
Forster and Calthorpe, 2000 [15]	NR	13	NR	2/11	Intertrochanteric fracture (13)	NR	Surgical repair	England	1 year
Verma et al., 2009 [18]	2000–2007	26	102 (100–105)	3/23	NR	3.7	Surgical repair	England	NR
Ogawa et al., 2021 [27]	2010–2018	4093	101.2 ± 1.46	434/3659	Femoral neck fracture (1252), trochanteric (2730), subtrochanteric (112)	2597 waited < 3 days, 1496 waited > 3 days	Surgical repair	Japan	NR
Manoli et al., 2017 [29]	2000–2011	1150	101.3 ± 1.8	143/1007	Intertrochanteric (661), transcervical (245), unspecified neck fracture (163), isolated greater trochanter fracture (41), subtrochanteric (38), open fracture (2)	1.7 ± 2.0	NR	USA	NR
Langenhan et al., 2022 [7]	2006–2020	85	(100–106)	14/71	Intracapsular (37), extracapsular (48)	0.8 (0.1–10.3)	Surgical repair	Germany	1 year
Blanco et al., 2020 [26]	2009–2018	48	101.4 ± 1.34	7/41	Intracapsular (26), extracapsular (22)	3.1 ± 2.3	Surgical repair	Spain	1 year
Buchanan et al., 2016 [21]	2000–2012	32	101.7 (100–105)	4/28	Intracapsular (14), intertrochanteric (16), subtrochanteric (2)	NR	Surgical repair	NR	4 months

*NR* not reported

### Study quality assessments

All studies identified were case series or cross-sectional studies, with a corresponding level of evidence being four. Whether relevant outcome measures were established a-[ priori or if outcome assessors were blinded to intervention administered were unclear for all studies (Table 2). Overall, the majority of studies included exhibited methodological limitations pertaining to low level of evidence and concerns regarding risk of bias.

**Table 2:** Results of risk of bias assessment

Case series quality appraisal checklist (IHE, 2014) risk of bias assessment questions	López-Torres et al., 2020 [4]	Morice et al., 2017 [25]	Dick et al., 2017 [32]	Shabat et al., 2004 [17]	Barrett-Lee et al., 2021 [33]	Moore et al., 2017 [24]	Cheung et al., 2017 [23]	Langenhan et al., 2022 [7]
Was the hypothesis/aim/objective of the study clearly stated?	Yes	Yes	Yes	Yes	Yes	Yes	Yes	Yes
Was the study conducted prospectively?	No	No	No	No	No	No	Unclear	No
Were the cases collected in more than one centre?	No	No	No	No	Yes	No	Yes	Yes
Were patients recruited consecutively?	No	No	No	No	No	No	Yes	No
Were the characteristics of the patients included in the study described?	Yes	Yes	Yes	Yes	Yes	Yes	Yes	Yes
Were the eligibility criteria (i.e., inclusion and exclusion criteria) for entry into the study clearly stated?	No	Yes	No	No	No	Yes	Partial	Yes
Did patients enter the study at a similar point in the disease?	Yes	Yes	Yes	Yes	Yes	Yes	Yes	Yes
Was the intervention of interest clearly described?	Yes	No	No	No	No	No	No	Yes
Were additional interventions (co-interventions) clearly described?	Yes	No	No	No	No	No	No	Yes
Were relevant outcome measures established a priori?	Unclear	Unclear	Unclear	Unclear	Unclear	Unclear	Unclear	Unclear
Were outcome assessors blinded to the intervention that patients received?	Unclear	Unclear	Unclear	Unclear	Unclear	Unclear	Unclear	Unclear
Were the relevant outcomes measured using appropriate objective/subjective methods?	Yes	Yes	Yes	Yes	Yes	Yes	Yes	Yes
Were the statistical tests used to assess the relevant outcomes appropriate?	Yes	Yes	No	Yes	NA	Not applicable	Yes	Yes
Was follow-up long enough for important events and outcomes to occur?	Yes	Yes	Yes	Yes	Yes	Yes	Yes	Yes
Were losses to follow-up reported?	Yes	Yes	No	No	No	No	No	Yes
Did the study provide estimates of random variability in the data analysis of relevant outcomes?	No	Yes	No	Yes	No	No	Yes	Yes
Were the adverse events reported?	No	Yes	Yes	Yes	Yes	No	No	Yes
Were the conclusions of the study supported by results?	Yes	Yes	Yes	Yes	Yes	Yes	Yes	Yes
Were both competing interests and sources of support for the study reported?	No	Yes	Yes	No	No	Yes	Yes	Yes
Risk of bias assessment	High	Some concerns	High	High	High	High	Some concerns	Low

Appraisal tool for cross-sectional studies (Downes et al., 2016) risk of bias assessment questions	Oliver and Burke, 2004 [16]	Sarasa-Roca et al., 2022 [28]	Mazzola et al., 2016 [22]	Mosfeldt et al., 2019 [31]	Verma et al., 2009 [18]	Ogawa et al., 2021 [27]	Manoli et al., 2017 [29]	Blanco et al., 2020 [26]	Buchanan et al., 2016 [21]
Were the aims/objectives of the study clear?	Yes	Yes	Yes	Yes	Yes	Yes	Yes	Yes	Yes
Was the study design appropriate for the stated aim(s)?	Yes	Yes	Yes	Yes	Yes	Yes	Yes	Yes	Yes
Was the sample size justified?	No	No	No	No	No	No	No	No	No
Was the target/reference population clearly defined? (Is it clear who the research was about?)	Yes	Yes	Yes	Yes	Yes	Yes	Yes	Yes	Yes
Was the sample frame taken from an appropriate population base so that it closely represented the target/reference population under investigation?	Yes	Yes	Yes	Yes	Yes	Yes	Yes	Yes	Yes
Was the selection process likely to select subjects/participants that were representative of the target/reference population under investigation?	Yes	No	No	No	Yes	No	Yes	No	No
Were measures undertaken to address and categorise non-responders?	No	No	No	No	No	No	No	Yes	No
Were the risk factor and outcome variables measured appropriate to the aims of the study?	Yes	Yes	Yes	Yes	Yes	Yes	Yes	Yes	Yes
Were the risk factor and outcome variables measured correctly using instruments/measurements that had been trialled, piloted or published previously?	Yes	Yes	Yes	Yes	Yes	Yes	Yes	Yes	Yes
Is it clear what was used to determined statistical significance and/or precision estimates? (e.g. p-values, confidence intervals)	Yes	Yes	Yes	Yes	Yes	Yes	Yes	Yes	Yes
Were the methods (including statistical methods) sufficiently described to enable them to be repeated?	No	Yes	Yes	Yes	Yes	Yes	Yes	Yes	No
Were the basic data adequately described?	Yes	Yes	Yes	Yes	Yes	Yes	Yes	Yes	Yes
Does the response rate raise concerns about non-response bias?	Not reported	Not reported	Not reported	Not reported	Not reported	Not reported	Not reported	No	Not reported
If appropriate, was information about non-responders described?	Not reported	Not reported	Not reported	Not reported	Not reported	Not reported	Not reported	No	Not reported
Were the results internally consistent?	Yes	Yes	Yes	Yes	Yes	Yes	Yes	Yes	Yes
Were the results presented for all the analyses described in the methods?	Yes	Yes	Yes	Yes	Yes	Yes	Yes	Yes	Yes
Were the authors' discussions and conclusions justified by the results?	Yes	Yes	Yes	Yes	Yes	Yes	Yes	Yes	Yes
Were the limitations of the study discussed?	Yes	Yes	Yes	Yes	Yes	Yes	Yes	Yes	Yes
Were there any funding sources or conflicts of interest that may affect the authors’ interpretation of the results?	Not reported	No	Not reported	No	No	No	No	No	Not reported
Was ethical approval or consent of participants attained?	Not reported	Not reported	Not reported	Not required	Not reported	Not required	Yes	Not required	Yes
Risk of bias assessment	High	Some concerns	High	Some concerns	Some concerns	High	Low	High	High

### Mortality outcomes of hip fractures in centenarians

**Fig. 2:**
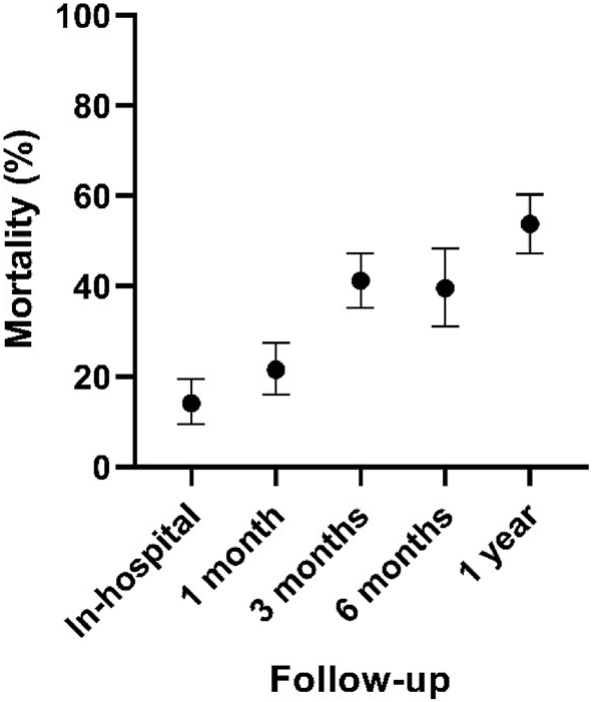
Pooled overall mortality following a hip fracture

**Fig. 3:**
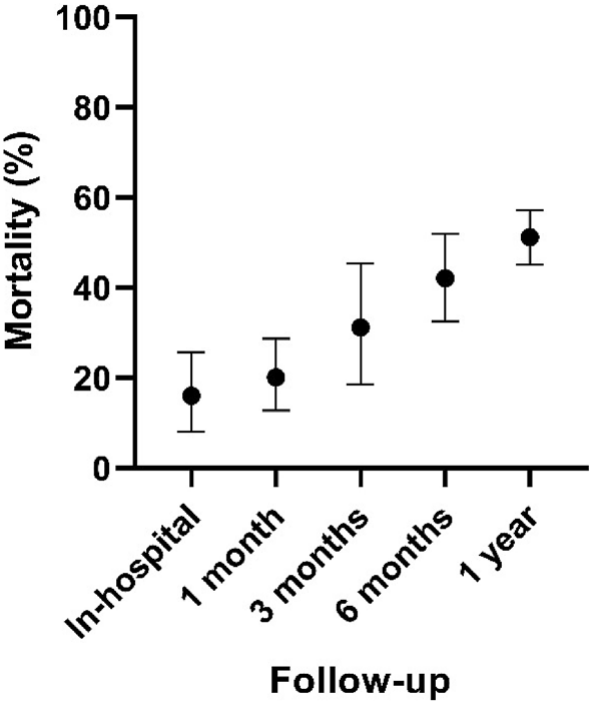
Pooled mortality in patients undergoing surgical intervention

Mortality was assessed in all 23 included studies (Appendix 2). One-year mortality was 53.8% (*n* = 1341). Mortality following a hip fracture was 14.1% (*n* = 5695) in-hospital (Table 3, Fig. 2). A sub-group analysis of surgical management of hip fractures was also performed. This included 16 studies (*n* = 4875) [4, 7, 15–28]. One-year mortality following surgical intervention was 51.2% (*n* = 706). In-hospital mortality was 16.0% (*n* = 4464) (Table 4***, ***Fig. 3).

**Table 3:** Pooled overall mortality following a hip fracture

	Mortality (%)	LCI (%)	HCI (%)	Cochran’s *Q*	Higgins *I*^2^ (%)	*N*
In-hospital	14.1	9.5	19.5	134.5	90.3	5695
1 month	21.5	16.1	27.4	67.3	77.7	1313
3 month	41.2	35.2	47.3	12.0	41.7	782
6 months	39.5	31.0	48.4	26.6	69.9	576
1 year	53.8	47.2	60.3	64.3	76.7	1341

*LCI* lower 95% confidence interval, *HCI* higher 95% confidence interval, *N* number of patients included in analysis

**Table 4:** Pooled mortality in patients undergoing surgical intervention

	Mortality (%)	LCI (%)	HCI (%)	Cochran’s *Q*	Higgins *I*^2^ (%)	*N*
In-hospital	16.0	8.1	25.7	102.2	90.2	4464
1 month	20.1	12.8	28.7	35.1	71.5	426
3 month	31.2	18.6	45.4	7.2	58.6	151
6 month	42.1	32.5	52.0	25.3	72.4	541
1 year	51.2	45.2	57.2	21.7	49.3	706

*LCI* lower 95% confidence interval, *HCI* higher 95% confidence interval, *N* number of patients included in analysis

### Secondary outcomes

#### Complications following hip fractures in centenarians

Nine studies (*n* = 340) [4, 7, 16, 17, 19, 28, 30, 32, 33] reported data on the number of patients exhibiting complications for meta-analysis (Appendix 3). Pooled complication rate was 50.5%. Six studies (*n* = 4244 patients) [17, 25, 27, 28, 32, 33] presented data on post-operative complications. Pneumonia was the most common complication following treatment of hip fracture in centenarians. This occurred in 10.0%. This was followed by urinary tract infection (3.7%), arrhythmia (3.2%), heart failure (2.9%), anaemia (2.8%) and intensive care unit admission (2.3%). Other complications had an incidence of less than 2% (Table 5).

**Table 5:** Pooled complications following treatment for hip fractures in centenarians

Complication	Prevalence (%)	LCI (%)	HCI (%)	Cochran’s *Q*	Higgins *I*^2^ (%)
Pneumonia	10.0	0	27.0	104.1	95.2
Urinary tract infection	3.7	0	10.8	42.2	88.1
Arrhythmia	3.2	0	8.7	29.9	83.3
Heart failure	2.9	0.6	6.7	12.3	59.4
Anaemia	2.8	0	8.8	38.2	86.9
Intensive care unit admission	2.3	1.0	4.0	6.1	17.9
Acute Kidney Injury	2.0	0	6.0	24.8	79.8
Unspecified respiratory complications	1.9	0.5	4.1	7.4	32.2
Early prosthetic dislocation	1.6	0	5.1	20.8	76.0
Coronary heart disease	1.6	1.2	2.0	0.9	0.0
Respiratory insufficiency	1.4	0	4.7	21.0	76.2
Surgical site infection	1.3	0	4.2	16.7	70.1
Pulmonary oedema	1.0	0	3.2	12.9	61.3
Stroke	0.9	0.6	1.2	0.1	0
Delirium	0.8	0	2.8	12.4	59.6
Pyelonephritis	0.8	0	2.8	12.4	59.6
Myocardial infarction	0.8	0	2.8	12.6	60.4
Wound haematoma	0.7	0.1	1.9	6.1	18.3
Transient ischaemic attack	0.5	0	1.7	8.4	40.6
Gastrointestinal bleed	0.5	0	1.7	8.4	40.6
Renal failure	0.5	0.3	0.7	0.3	0
Portal hypertension	0.5	0	1.7	8.5	41.4
Deep vein thrombosis	0.5	0	1.7	8.6	41.5
Intra-op cardiac arrest	0.5	0	1.7	8.6	41.5
Pulmonary embolism	0.4	0.2	0.6	0.5	0

*LCI* lower 95% confidence interval, *HCI* higher 95% confidence interval

#### Comorbidities in centenarians with a hip fracture

A total of ten studies (n = 5158 patients) [4, 7, 16–18, 27, 28, 31, 33, 34] reported on the number and type of comorbidities observed and presented data to be included in the meta-analysis (Appendix 4).

Dementia was the most commonly reported comorbidity (26.2%), followed by hypertension (15.6%), diabetes (5.5%), and cancer (3.7%). Unspecified cardiovascular disease had a pooled prevalence of 7.5%, but studies reporting this comorbidity did not specify what pathology was observed [7, 17, 33] (Table 6).

**Table 6:** Pooled comorbidities in centenarians with a hip fracture

Comorbidity	Prevalence (%)	LCI (%)	HCI (%)	Cochran’s *Q*	Higgins *I*^2^ (%)
Dementia	26.2	15.7	38.2	279.9	96.8
Hypertension	15.6	3.4	33.1	715.7	98.7
Cardiovascular disease (unspecified)	7.5	1.00	18.2	433.6	97.9
Diabetes	5.5	1.9	10.7	139.2	93.5
Cancer	3.7	1.4	6.9	76.4	88.2
Heart failure	3.5	0	10.6	446.5	98.0
Cerebrovascular disease	3.4	1.0	7.2	104.5	91.4
Pulmonary disease	2.3	0.9	4.3	42.2	78.7
Renal disease	1.9	0.1	5.00	110.5	91.9
Coronary heart disease	1.7	0	4.6	140.7	93.6
Audio visual impairment	1.2	0	3.8	100.5	91.0
Peptic ulcer	1.1	0.2	2.5	37.1	75.7
Parkinson's disease	1.0	0	2.8	62.8	85.7
Osteoarthritis	0.8	0	2.5	67.4	86.6
Anaemia	0.7	0	2.4	103.1	91.3
Peripheral vascular disease	0.6	0.4	0.8	5.4	0.0
Atrial fibrillation	0.6	0	1.8	45.6	80.3
Rheumatoid arthritis	0.4	0	1.00	23.9	62.4
Liver disease	0.1	0	0.2	3.5	0.0
Connective tissue disease	0.1	0	0.2	2.8	0.0
Depression	0.1	0	0.2	10.2	11.9

*LCI* lower 95% confidence interval, *HCI* higher 95% confidence interval

### Centenarians vs. non-centenarians: co-morbidities

Three studies presented data on comorbid disease prevalence in centenarians and non-centenarians who sustained a hip fracture [26, 29, 31]. Mosfeldt et al. [31] reported centenarians with a hip fracture had a lower median CCI than patients aged 70–99 years with a hip fracture (0 vs 1, *P* < 0.001). In addition, centenarian were less likely to suffer from renal disease (*P* = 0.01), congestive heart failure (*P* = 0.003), diabetes mellitus (*P* = 0.005), cerebrovascular disease (*P* = 0.002), peripheral vascular disease (*P* < 0.001), pulmonary disease (*P* < 0.001), ulcer disease (*P* = 0.04), malignancy (*P* < 0.001), rheumatic disorders (*P* = 0.002), dementia (*P* < 0.001), and less likely to use anti-osteoporotic medications (*P* < 0.001). There were non-statistically significant differences between groups in number of patients with liver disease, myocardial infarction, paralysis, and fracture type. In addition, Blanco et al. [26] found centenarians had a lower incidence of cognitive impairment (*P* < 0.001) and were taking a lower number of medications (*P* = 0.034) than patients aged 80–99 years.

It was possible to pool data comparing CCI between patients aged 65–99 years (*n* = 167,065) and centenarians (*n* = 1198) in two studies [26, 29]. Charlson Comorbidity Index was significantly lower in centenarians (mean difference − 0.27; 95% CI − 0.11 to − 0.43; *I*^2^ 81.3%).

## Discussion

Knowledge of the mortality associated with hip fracture provides further evidence of the importance of health professionals to hold informed discussions with patients and relatives regarding prognosis following this injury type. However, the majority of studies included in this review exhibited methodological limitations pertaining to low level of evidence and concerns regarding risk of bias. Caution should, therefore, be placed when interpreting these findings.

Due to increasing mortality with age, it is important to ascertain whether hip fractures lead to increased mortality in centenarians. A single study found centenarians with a hip fracture had a lower mean survival time and hospitalization-free survival time than those without a hip fracture [22]. Further research should perform such analysis to more reliably establish the disease-specific mortality associated with hip fracture. Alvarez et al. [35] stratified mortality in centenarians according to degree of frailty. “Robust” and “intermediate” centenarians (higher cognitive and physical capabilities than frail centenarians) had a mortality of less than 10% within a year. A pooled 1-year mortality of 51% was calculated in this meta-analysis. Therefore, centenarians with hip fracture may experience a higher mortality than centenarians without hip fracture. Further research matching centenarians with hip fracture and autonomous centenarians without a hip fracture is required to validate this hypothesis.

Current evidence suggests centenarians suffering from a hip fracture have a higher pre-injury functional status [30], lower medication burden [26, 30], and a lower number of comorbidities [26, 29, 31] than their younger peers (with the latter demonstrated by meta-analysis). Individuals suffering from hip fractures are more likely to be ambulatory [7]. This may render centenarians suffering from hip fractures a self-selecting group, with a lower comorbidity burden allowing them to ambulate, and subsequently fall. This may explain the lower prevalence of comorbidities when compared to a random selection of older people [33]. However, we can only draw conclusions based on our study population (centenarians with hip fractures). Our findings are not applicable to centenarians without hip fractures. In addition, they are hindered by the low level of evidence of the studies included in this review.

Dementia was the most commonly reported comorbidity, being present in over a quarter of patients suffering from a hip fracture (26.2%). This is lower than the estimated 40% prevalence in patients over 100 years of age [36]. This may be explained by the fact that centenarians with moderate-severe dementia are mostly dependent on a wheelchair for ambulation, and are hence less susceptible to falls and hip fractures. Hypertension was the second most common comorbidity, with a pooled prevalence of 15.6%. Antihypertensive medication use may predispose fall injuries among older people [37]. Mazzola et al. [22] matched centenarians with and without a hip fracture, and found the former were more likely to be on an antihypertensive agent. However, whether hypertension itself or taking antihypertensive medications are the culprits remains unclear. A lower prevalence of hypertension was noted in this meta-analysis than is usually reported in other population-based studies [38]. Centenarians with multiple morbidities, requiring multiple medications like anti-hypertensives, are more likely to be less ambulatory. Therefore, hypertension as a comorbidity may be under-represented in our study population. Other medications which may increase the risk of falls in the elderly include diuretics, sedatives, hypnotics, antidepressants, benzodiazepines and nonsteroidal anti-inflammatory drugs [39]. Though medication reviews are commonly performed in clinical practice regardless of patient age, these are particularly relevant for centenarians given the detrimental outcomes resulting from fractures.

Pneumonia was the most common complication following treatment of hip fracture in centenarians, occurring in 10.0% of patients. This was followed by urinary tract infection (3.7%). Infective presentations could be attributed to centenarians with hip fractures having a long hospital stay (calculated at 28.95 days on average). Considering infection were the two most common complications, antibiotic prophylaxis in centenarians with a hip fracture may be warranted. Other common complications included arrhythmia (3.2%), heart failure (2.9%), and anaemia (2.8%). These may prompt cardiovascular input. Cases of hip fracture in centenarians are typically complex, with potentially multiple comorbidities and complications. A multidisciplinary approach is required for optimal treatment, with orthogeriatricians’ involvement advocated by European and UK guidelines [40, 41].

Sub-group analysis comparing overall mortality irrespective of treatment, and mortality in patients undergoing surgical repair indicated similar survival and overlapping confidence intervals up to 1 year after the hip fracture. Therefore, surgical repair following a hip fracture may not provide an additional benefit in terms of mortality reduction. A fixed fracture may lead to improved pain management, quality of life, ease of nursing and personal care, which would justify operative intervention. This meta-analysis cannot determine whether surgical repair yields better functional outcomes or lower complication rates than conservative management due to the lack of studies evaluating these parameters in operative and conservative interventions separately. Further study should explore these parameters to ascertain whether non-operative treatment leads to increased functional recovery.

Time-to-surgery was reported in 11 studies. These ranged from 1.6 to 4 days. This is in contrast with current treatment guidelines, which recommend surgery be performed within 24 h of admission [40, 41]. Such delay could be explained by the need for medical optimisation before orthopaedic surgery can be performed on centenarians [42]. However, lack of capacity in the operating theatre and/or ageism may be contributing factors [43]. Only one study reported that, of nine patients, seven were operated within 48 h, whereas the remaining two were delayed due to medical stabilisation. Further work on hip fractures in centenarians should report reasons for delays in time to surgery, given this is a negative prognostic indicator and must be addressed [44].

Our review was limited by the inclusion of studies with varying dates and duration of retrospective periods. As surgical procedures may have changed over the course of long time periods, the mortality data may be affected. In addition, to calculate mortality regardless of treatment, patients who did not undergo surgical repair were included. These patients may have had severe comorbidities or have been at the end of life, which could have increased the mortality figures calculated. Current evidence has limitations which must be improved to increase the understanding of outcomes of fractures in centenarians. Firstly, the vast majority of studies carried a low level of evidence with major concerns regarding their risk of bias. All studies were case series, which is the most feasible study design to evaluate outcomes of fractures in centenarians given the small number of patients reaching this age. To conduct large clinical trials of this population, a national or international network of trial centres would be warranted. Secondly, only two studies reported patient ethnicity [23, 27]. Studies also poorly reported the characteristics of their cohort in relation to concomitant injuries and osteoporotic status. Given that both factors significantly impact on prognosis, this is an important limitation which should be addressed in future study reporting on this population. Thirdly, no studies clarified the time-frame following fracture within which complications were identified. Therefore, it is not possible to determine when these occur following hip fractures in centenarians. Fourthly, 12 studies did not report time-to-surgery. This is a crucial parameter for successful operation and survival, and is particularly important for prognosis in centenarians [44]. Further research should report on this parameter given its impact on prognosis. Finally, there was a very low number of studies reporting outcomes for surgical and conservative management separately. The comparison of these approaches, investigating both mortality but also health-related quality of life, is required to improve understanding on what is the best care for people aged 100 years and older who experience hip fracture.

## Conclusion

Hip fractures in centenarians typically involve complex patient presentations with diverse comorbidities. Dementia, hypertension and diabetes are the most common comorbidities in centenarians with a hip fracture. However, the majority of studies included in this review exhibited methodological limitations pertaining to low level of evidence and concerns regarding risk of bias. Therefore, it is difficult to give a more precise description of mortality and comorbidities in centenarians. Effective cross-discipline communication and discussion with patients and carers regarding higher mortality rates are advised.

## Appendix 1: search strategy

Trauma OR orthopaedics OR injury OR fracture.

AND

Centenarian* OR 100 years old.

AND

Outcome* OR discharge OR disab* OR mortality OR management OR treatment OR predisposing OR risk factors OR complication* OR comorbidities OR length of stay.

*Deduplicate*

## Appendix 2: raw data—mortality following hip fractures in centenarians

**Table:**

Study	Mortality
Oliver and Burke, 2003 [16]	11.1% in-patient, 33.3% 1-month, 50% 4-month
López-Torres et al., 2020 [4]	13.8% in-patient, 21.5% 1-month, 54.2% 1-year
Morice et al., 2017 [25]	7.7% in-hospital, 33.3% 3-month, 42.1% after the 1-year
Sarasa-Roca et al., 2022 [28]	33.3% in-patient, 41.7% 1-month, 62.5% 6-month, 66.7% 1-year
Dick et al., 2017 [32]	30% in-hospital, 30% 1-month, 39% 3-month, 50% 6-month, 77% 1-year
Mazzola et al., 2016 [22]	38.8% 6-month, 51.2% 1-year
Mosfeldt et al., 2019 [31]	34% 1-month, 49% 3-month, 66% 1-year
Shabat et al., 2004 [17]	non-op: 75% 1-month, 100% 2-month. Surgical: 5.3% 1-month, 42.1% 6-months, 42.1% 1-year
Barrett-Lee et al., 2021 [33]	27% 1-month, 40% 3-month, 55% 1-year
Moore et al., 2017 [24]	22% in-patient, 22% 1-month, 71% 1-year
Bermejo Boixareu et al., 2020 [34]	20.16% 1-month
Hogan et al., 2019 [45]	12.3% in-patient
Ng and Kwek, 2015 [20]	Surgical: 0 1-month, 0 3-month, 16.7% 6-month, 33.3% 1-year. non-op: 14.3% 1-month, 28.6% 3-month, 42.8% 6-month, 57.2% 1-year
Tarity et al., 2013 [19]	operative: 15% in-patient, 20% 1-month, 30% 3-month, 45% 6-months, 60% 1-year. Non-op: 100% 3-month
Barceló et al., 2018 [30]	41.4% 3-month, 62.1% 1-year
Cheung et al., 2017 [23]	8% 1-month, 25% 6-month, 37% 1-year
Forster and Calthorpe, 2000 [15]	31% 1-month, 50% 6-month, 56% 1-year
Verma et al., 2009 [18]	17.3% in-patient
Ogawa et al., 2021 [27]	3.2% in-patient
Manoli et al., 2017 [29]	7.4% in-patient
Langenhan et al., 2022 [7]	18.8% in-patient, 27.1% 1-month, 42.4% 3-month, 55.3% 6-month, 61.2% 1-year
Blanco et al., 2020 [26]	8.3% in-patient, 8.9% 1-month, 40% 1-year
Buchanan et al., 2016 [21]	34.4% in-patient, 31.3% 1-month, 62.5% 4-month

## Appendix 3: raw data—complications observed in centenarians with a hip fracture

**Table:**

Study	Complication rate	Complications observed
Oliver and Burke, 2003 [16]	11.1%	Not reported
López-Torres et al., 2020 [4]	16.9%	Not reported
Morice et al., 2017 [25]	Not reported	Early dislocation of bipolar hip prothesis (3), surgical-site infection (2), heart failure (1), delirium (2), pneumonia (2), and pyelonephritis (2)
Sarasa-Roca et al., 2022 [28]	100%	Pneumonia (4), respiratory insufficiency (4), heart failure (5), anaemia (6), pulmonary oedema (1), arrhythmia (1), acute kidney injury (2), portal hypertension (1), prosthetic dislocation (1)
Dick et al., 2017 [32]	71%	Urinary tract infection (5), pneumonia (10), coronary heart disease (2), atrial fibrillation (2), deep vein thrombosis (1), cardiac arrest (1)
Shabat et al., 2004 [17]	21.7%	wound hematoma (2), urinary tract infection, arrhythmia (2)
Barrett-Lee et al., 2021 [33]	46.5%	urinary tract infection (3), acute kidney injury (3), anaemia (2), pneumonia (7), arrhythmia (1), gastrointestinal bleed (1), pulmonary oedema (1), surgical-site infection (1), transient ischaemic attack (1),
Tarity et al., 2013 [19]	43%	Not reported
Barceló et al., 2018 [30]	97%	Not reported
Ogawa et al., 2021 [27]	Not reported	respiratory complications (161), heart failure (120), coronary heart disease (65), stroke (35), pulmonary embolism (16), renal failure (19), intensive care unit admission (145), surgical site hematoma (22)
Langenhan et al., 2022 [7]	28.2%	Not reported

## Appendix 4: raw data—comorbidities in centenarians with a hip fracture

**Table:**

Study	Comorbidities (number of patients)
Oliver and Burke, 2003 [16]	Dementia (5), heart failure (5), breast cancer (1)
López-Torres et al., 2020 [4]	Hypertension (43), dementia (27), osteoarthritis (15), heart failure (14), cancer (13), cerebrovascular disease (11), type 2 diabetes mellitus (11), atrial fibrillation (10)
Sarasa-Roca et al., 2022 [28]	Dementia (6), stroke (4), coronary heart disease (1), cancer (1), depression (1)
Mosfeldt et al., 2019 [31]	heart failure (85), coronary heart disease (21), type 2 diabetes mellitus (11), cerebrovascular disease (50), peripheral vascular disease (6), pulmonary disease (12), peptic ulcer (18), cancer (23), rheumatoid arthritis (6), dementia (16), renal disease (2), liver disease (1),
Shabat et al., 2004 [17]	Hypertension (11), cardiovascular disease (8), type 2 diabetes mellitus (10), Parkinson's disease (13), dementia (15) audio visual impairment (17)
Barrett-Lee et al., 2021 [33]	heart disease (16), respiratory disease (4), chronic kidney disease (23), dementia (16)
Bermejo Boixareu et al., 2020 [34]	dementia (83)
Verma et al., 2009 [18]	Heart disease (6), pneumonia (1)
Ogawa et al., 2021 [27]	Coronary heart disease (55), heart failure (640), peripheral vascular disease (23), cerebrovascular disease (301), dementia (811), pulmonary disease (172), connective tissues disease (3), peptic ulcer (140), type 2 diabetes mellitus (267), renal disease (131), cancer (75), liver disease (3), hypertension (1627), coronary heart disease (331), Parkinson's Disease (16), anaemia (179)
Langenhan et al., 2022 [7]	Dementia (47), hypertension (60), type 2 diabetes mellitus (22), cardiovascular disease (58), cancer (12), pulmonary disease (5)
